# Multiple Routes to Control in the Prime-Target Task: Congruence Sequence Effects Emerge Due to Modulation of Irrelevant Prime Activity and Utilization of Temporal Order Information

**DOI:** 10.5334/joc.143

**Published:** 2021-03-10

**Authors:** David Dignath, Andrea Kiesel, Moritz Schiltenwolf, Eliot Hazeltine

**Affiliations:** 1 Eberhard Karls University of Tübingen, Germany; 2 University of Freiburg, Germany; 3 University of Iowa, US

**Keywords:** cognitive control, congruence sequence effect (CSE), Gratton-effect, Conflict adaptation, prime-target task

## Abstract

In interference tasks, the magnitude of the congruency effect is reduced in trials that follow an incongruent trial. This congruence sequence effect (CSE) reflects cognitive control processes, yet accounts disagree when and how control is exerted. Here, we address these questions in the context of the prime-target task. In this task, control can either modulate early prime or late target information. Furthermore, control can utilize information specific to the stimulus (perceptual features) or relational information between stimuli (temporal order). Two experiments (N = 41 | N = 62) were conducted using a prime-target task with arrows (prime) and letters (target). We presented either the prime before the target or the target before the prime. For both trial-type transitions, the CSE was assessed. Regarding the first question, when is control exerted, results showed a larger CSE for prime→target relative to target→prime trials. This suggests that control in the prime-target task modulates prime activity. Regarding the second question, how is control exerted, a combined analysis of both experiments showed a larger CSE for repetition of the same prime and target order across two trials (e.g., previous trial: prime→target; current trial: prime→target) compared to changes (e.g., previous trial: prime→target; current trial: target→prime), suggesting that control in the prime-target task can employ temporal selection.

## Introduction

Multitasking is a societal fact; people must engage in multitasking in work settings and they do so voluntarily in their leisure time. Yet, multitasking comes at a cost and often leads to worse performance than single-tasking (see Koch, Poljac, Müller, & Kiesel, 2018 for a review). A central problem of multitasking arises due to concurrently activated processes that interfere with one another (Allport, 1987; Navon & Miller, 1987). Therefore, it has been suggested that our cognitive system has evolved dedicated control mechanisms to manage interference (Botvinick et al., 2001; Miller & Cohen, 2001). Here, we address such control mechanisms by taking the example of the prime-target task to ask when and how control operates.

The present research is part of a collection of articles on multitasking. It contributes to this special issue by adopting a task-switching perspective to the study of interference control in single-tasking (Kiesel et al., 2010). In a typical single-task experiment, instructions specify a rule that describes how participants should respond to certain stimuli. Based on these instructions, participants form a task-set, a mental representation that links stimuli to responses. Task-sets allow the control of behavior according to these specified rules, for instance, by biasing attention towards relevant and away from irrelevant information (see Künzell et al., 2018; Schumacher & Hazeltine, 2016). However, despite such explicit (i.e., instructed) task-sets, participants may also form implicit task-sets and infer adaptive control policies based on covariations of stimulus, responses and contextual information (e.g., Badre, Kayser, & D’Esposito, 2010; Dreisbach, Goschke, & Haider, 2006). Here, we present a conceptual analysis of the prime-target task, an often-used paradigm to study interference control, showing that control can be attributed both to explicit (instructed) and implicit (inferred) task-sets. We then devised a procedure to test the relative contribution of explicit and implicit task-sets which is inspired by research on task-switching. Together, this part of our research suggests how multiple task-sets and resulting attentional control policies could control performance in single-tasking. In addition, we believe that a better understanding of interference control in general, will also inform research on multitasking – although interference produces severe costs during multitasking, it is anything but specific to multitasking. Rather it constitutes a general feature of a cognitive architecture that codes stimuli and responses in a distributed fashion (Cisek, 2007; Feng, Schwemmer, Gershman, & Cohen, 2014).

### Control of Interference: Empirical Evidence and Theoretical Models

In the lab, control is often studied with so-called interference tasks like Stroop, flanker, or the Simon task. For instance, in the prime-target task, which is also the focus of the present research, participants respond to the identity of a target that is preceded by a prime stimulus (Kunde, Kiesel, & Hoffmann, 2003; Pohl, Kiesel, Kunde, & Hoffmann, 2010; Vorberg, Mattler, Heinecke, Schmidt, & Schwarzbach, 2003). On congruent trials, prime and target afford the same response which facilitates responding. In contrast, on incongruent trials, prime and target afford different responses that impair responding. Performance differences between congruent and incongruent trials (i.e., congruency effect [CE]) can be explained by assuming that relevant target information and irrelevant prime information produce interference as they are processed. While CEs provide an intuitive index of the strength of interference, many researchers used the congruency sequence effect (CSE) to infer the amount of interference control. CSEs refer to the observation of reduced congruency effects in trials that follow an incongruent trial (flanker: Gratton Coles, & Donchin, 1992; Simon: Praamstra, Kleine, & Schnitzler, 1999; Stroop: Kerns et al., 2004; prime-target: Kunde, 2003). CSEs suggest that the recent experience of response conflict during incongruent trials is followed by an upregulation of control, attenuating subsequent interference and response conflict (see Egner, 2007 for a review).

Often, CSEs have been explained by cybernetic models like conflict monitoring (e.g., Botvinick et al., 2001). These models assume that response conflict is registered and serves as a learning signal for subsequent control implementation. More specifically, during an incongruent trial, attentional weights change in favor of task-relevant information and attenuate irrelevant information, while only little changes in attentional weights take place during congruent trials (but see Lamers & Roelofs, 2011; Compton, Huber, Levinson, & Zheutlin, 2012). As a consequence for the upcoming trial, attentional weights alleviate the impact of irrelevant information on response selection more strongly after a previously incongruent relative to a previously congruent trial (for recent versions of conflict monitoring, see Alexander & Brown, 2011; Brown & Braver, 2005; Dignath, Eder, Steinhauser, & Kiesel, 2020; Verguts & Notebaert, 2009).

### Interference Control in the Prime-Target Task

While these models offer a viable account for the CSE in general, the specifics of control implementation seem to differ across tasks. For instance, in Stroop-like tasks incongruent trials lead to a strengthening of task-relevant information (Egner & Hirsch, 2005), while in the Simon task incongruent trials lead to a weakening of task-irrelevant information (Stürmer, Leuthold, Soetens, Schröter, & Sommer, 2002; Stürmer, Redlich, Irlbacher, & Brandt, 2007). Indeed, a rich research tradition emphasizes the need to differentiate carefully between conflict on various levels (e.g., Schuch, Dignath, Steinhauser, & Janczyk, 2019; De Houwer, 2003; Liu, Banich, Jacobson, & Tanabe, 2004). This line of research suggests that different interference tasks tap into distinct control mechanisms (see Kornblum, Hasbroucq, & Osman, 1990). Further support for this more fine-grained view comes from studies showing that control exerted in one task rarely transfers to a different task (Braem, Abrahamse, Duthoo, & Notebaert, 2014; Egner, 2008) and that control effects show little consistency across tasks (Eisenberg et al., 2019; Stahl et al., 2014). Accordingly, we believe that it might be premature to generalize findings from Stroop or flanker to other interference tasks without further empirical tests. Therefore, we aim to elaborate in more detail on the structure of control in the prime-target task (Hazeltine, Lightman, Schwarb, & Schumacher, 2011; Jost, Wendt, Luna-Rodriguez, Löw, & Jacobsen, 2017; Kiesel, Kunde, Hoffman, 2006; Kunde & Wühr, 2006; Reuss, Desender, Kiesel, & Kunde, 2014; Wendt, Luna-Rodriguez, & Jacobsen, 2014).

An emphasis on this task is motivated both by practical and theoretical aspects. Practically, the prime-target task has become increasingly popular in recent research that employed confound minimized designs to rule out alternative explanations of CSEs in terms of direct stimulus repetitions and stimulus-response (S-R) binding (e.g., Schmidt & Weissman, 2014; Weissman, Jiang, & Egner, 2014; Braem et al., 2019; Kunde & Wühr, 2006). One reason for this popularity might be that the prime-target task presents optimized conditions to probe CSEs relative to other interference tasks. For instance, Weissman and colleagues (2014) systematically compared confound-minimized designs across different tasks and found that the CSE was larger in the prime-target task and related tasks in which the irrelevant information preceded the target information relative to tasks with simultaneous presentation of irrelevant and relevant information. Consequently, the majority of subsequent studies that employed a confound-minimized design used the prime-target task (e.g., Dignath, Berger, Spruit, & van Steenbergen, 2019; Lim & Cho, 2018; Larson, Clayson, Kirwan, & Weissman, 2016; Jiang, Brashier, & Egner, 2015; Schröder, Dignath, & Janczyk, 2019). Theoretically, the prime-target task is interesting because it is often set-up to enable control on two dimensions. First, in many versions of the prime-target task, prime and target can be differentiated according to specific features like size, location, color, or shape of the stimuli. Thus, in these cases, control can be based on a selection mechanism that considers specific features of the stimulus. Second, unlike Stroop or flanker tasks, in the prime-target task stimuli are presented sequentially, separating irrelevant and relevant information in time. Accordingly, control can also recruit a selection mechanism that considers the temporal order of events.

Against this background, the present research critically revaluates the locus of control in the prime-target task and asks *how* and *when* control is exerted. Regarding the former question, accounts suggest that control can utilize information specific to the stimulus (perceptual features) or relational information between stimuli (temporal order). Regarding the latter question, different accounts suggest that control can either modulate irrelevant prime or relevant target information. In the next part, we will first review relevant literature on the question of how control is implemented before we turn to the question when control is implemented.

### How Control Is Implemented: Contrasting Feature- and Order-Based Control

Some authors have suggested that the temporal separation between prime and target stimuli modulate feature-based selection, for instance, by affecting the time course of S-R translations (Ridderinkhof, 2002; Burle, Possamaï, Vidal, Bonnet, & Hasbroucq, 2002). However, others have suggested that this additional temporal information about prime and target stimuli allows for a completely different selection mechanism (for reviews, see Coull, 2004; Nobre & Van Ede, 2018). Accordingly, control is based on a selection mechanism that considers relational information between prime and target, specifying the temporal order of relevant and irrelevant information.

Evidence for this account comes from a version of the prime-target task in which both prime and target share the same physical features and participants are instructed to select targets over distractors according to their relative temporal order (Hazeltine, 2011; see also Wendt, Kiesel, Geringswald, Purmann, & Fischer, 2014). Importantly, such a task design eliminates any selection mechanism based on stimulus features, because prime and target are drawn from the same set of stimuli. Instead, selection processes must rely on the temporal order of the two stimuli. Critically and in contrast to perceptual features like color or location, temporal order is not considered an inherent feature of the stimulus because it is not a property of the stimulus per se but rather emerges from its relationship to other stimuli within the context of the task (Hazeltine et al., 2011). Since there are no perceptual features that can be modulated to bias activation of the target over the prime, control based on temporal order selection may be different from control based on stimulus feature selection. Interestingly, because the CSE in the study by Hazeltine and colleagues (2011) reflects control processes that are defined in relation to parameters of a task-set, switching between different task-sets should abolish the CSE (see Grant, Cookson, & Weissman, 2020). Indeed, this is exactly what was found by Hazeltine and colleagues, showing that CSE based on temporal order selection critically depended on the repetition of the same task-set across trials (Hazeltine et al., 2011).

To summarize, previous research has provided evidence for temporal control when participants are instructed to use stimulus order and when stimulus configuration rendered selection based on perceptual features impossible. Here we ask whether temporal order control is used even under less optimal conditions, e.g. when both instructions and stimulus configurations afford selection based on perceptual features. According to the task-switching perspective introduced at the beginning, participants may spontaneously adopt an implicit task-set that considers temporal order information, possibly because temporal order is very salient in the prime-target task due to sequential presentation of stimuli. Thus, participants might use temporal order control as a default even under conditions in which this selection strategy is nominally irrelevant (e.g., because the explicitly instructed task-set refers to perceptual features). One aim of the present research is to test this *temporal control as default hypothesis*.

### When Control Is Implemented: Contrasting Distractor- and Target-Based Control

Theoretical accounts make different predictions regarding the point in time when control is exerted. For instance, according to the activation-suppression hypothesis, control influences response tendencies instigated by the prime (Ridderinkhof, 2002). In line with dual-route models, the irrelevant prime activates an unconditional route that competes with a conditional, target-related route (Kornblum et al., 1990). Control influences the unconditional route and is assumed to be time-consuming, resulting in initially strong activation of the unconditional route which becomes gradually suppressed over time. Furthermore, control persists across trials, leading to increased suppression of the unconditional route after previously incongruent relative to congruent trials (Ridderinkhof, 2002, Hazeltine, Akcay, & Mordkoff, 2011; see also Stürmer et al., 2002).

Relatedly, the response expectancy hypothesis also attributes control implementation to the prime (Weissman, Egner, Hawks, & Link, 2015). While it is assumed that the prime has the potential to activate response tendencies (see Weissman, 2019), this depends on the previous trial congruency. If the previous trial was congruent, the prime should activate the indicated response; however, if the previous trial was incongruent, the prime should activate the response opposite to the one afforded by the prime. Thus, control is thought to reflect biased expectations whether to ‘trust’ or ‘distrust’ the prime in the current trial based on previous trial congruency. Critically, both accounts agree that what needs to be controlled in the prime-target task is the distractor information in the prime and not the target.

An alternative to these views is suggested by the conflict monitoring model which proposes that control increases activation of the target (Botvinick et al., see also Verguts & Notebaert, 2008). More specifically, it is assumed that the currently relevant processing route becomes more strongly activated after an incongruent trial relative to a congruent trial, changing the relative weighting of relevant and irrelevant information in favor of the target stimulus. This model received empirical support from neuroimaging studies showing increased activity in perceptual areas for target-relevant information after a previously incongruent relative to congruent trial (Egner & Hirsch, 2005; Polk, Drake, Jonides, Smith, & Smith, 2008; Purmann & Pollmann, 2015). However, these studies employed Stroop-like tasks, making it unclear whether results can be transferred to the prime-target task.

Hence, another goal of the current research is to identify when control implementation takes place in the prime-target task. For that reason, we contrasted conditions that activate the distractor or the target first. Distractor-based control accounts make strong predictions about the temporal dynamics of control, including that an earlier presentation of the prime (distractor) will increase the chances of successful response suppression. In contrast, target-based control accounts do not make these predictions. Rather, according to our reading of these models, amplification of target processing should be independent of the time between distractor and target.

### Empirical Evidence for Distractor-Based Control or Temporal Order Selection?

A recent study by Weissman and colleagues (2015) directly manipulated the time between prime and target. In a first experiment, participants responded to the target while in half of the blocks the prime was presented before the target (sequential presentation mode), whereas in the other half of the blocks the prime was presented together with the target (simultaneous presentation mode). The CSE was larger in the sequential relative to simultaneous condition, suggesting that control is facilitated by pre-activation of the prime. However, this interpretation confounded the number of control dimensions in the prime-target task (selection by stimulus features and selection by temporal order) with the comparison of distractor and target-based control. While sequential trials allowed for selection based on stimulus features and temporal order information, simultaneous trials allowed only for selection based on stimulus features, but not temporal order. Thus, one could speculate that sequential trials showed a larger CSE not only because distractor information was presented before the target, but also because the CSE reflected the contribution of an additional control mechanism that was not applicable in simultaneous trials. Furthermore, in a second experiment, the authors intermixed sequential and simultaneous trials within blocks of trials. Interestingly, results showed two additional interaction effects. First, the CSE was generally larger for trials in which the presentation mode (sequential vs. simultaneous) repeated across trials. Second, the CSE was specifically larger for repeating trials with sequential compared to simultaneous presentation mode.

According to the authors, the first effect indicated that repeating the same context or task-set facilitated the CSE, suggesting that “the task representation and/or episodic retrieval view might partially (although not completely) explain the CSEs” (Weissman et al., 2015, p. 16). However, why was the repetition benefit of presentation modes larger for CSEs in sequential relative to simultaneous trials? One explanation, favored by the authors, was that distractor-based control requires “processing the distracter before the target to a high degree” (Weissman et a., 2015, p. 15). However, an alternative interpretation would assume that this effect is the result of a combination of two control mechanisms in sequential trials. Of these two control mechanisms, selection by temporal order requires an activated task-set that is impaired for task switches relative to repetitions. These speculations provide another motivation for the present work to study control mechanisms in the prime-target task in more detail.

## The Present Research

For a further characterization of control in the prime-target task, we pose two related questions concerning the structure of control. First, we ask when control is exerted, i.e., whether control is rather concerned with properties of the prime or the target. Second, we ask how control is exerted, i.e., whether control employs selection by temporal order even if participants are instructed to select targets according to perceptual features.

To answer these questions, two experiments were conducted using a prime-target task (Exp. 1a was run in the lab, Exp. 1b was a close replication run online). We used primes and targets drawn from separate sets of clearly distinguishable stimuli so that the target could be identified without relying on temporal order. We manipulated the temporal order of prime and target in both directions symmetrically – in half the trials, the prime was presented before the target (prime→target trials), while the other half, the target was presented before the prime (target→prime trials). This manipulation has the advantage over the sequential/simultaneous trials used in Weissman et al. (2015) that both trial types allow for control based on specific features of the stimuli and for control based on the temporal order of stimuli. However, a possible caveat could be that CEs differ markedly between trial types, for instance, because target→prime trials do not elicit a CE at all. To take precautions against this possible limitation, we used letters as target stimuli (D, G, H, or S), but arrows as prime stimuli. Participants were instructed to respond to the identity of a target letter (D, G, H, or S) by pressing one of the four possible arrow keys of the keyboard. Previous research showed that arrow stimuli trigger reflexive attention (Eimer, 1997; Hommel, Pratt, Colzato, & Godijn, 2001) and cause fast and automatic response tendencies within 200 ms after stimulus presentation (Eimer, 1995; Verleger, Vollmer, Wauschkuhn, van der Lubbe, & Wascher, 2000). Therefore, we reasoned that arrow primes should influence the target response even for target→prime trials. Arrows pointed either upwards, downwards, to the left, or the right, so that the arrow distractor was either congruent or incongruent to the response. To avoid low-level feature binding effects (e.g., direct repetitions, negative priming or partial repetition costs, see Mayr, Awh, & Laurey, 2003, Davelaar & Stevens 2009; Hommel, Proctor, & Vu, 2004) we used two different sets of targets and primes (i.e., vertical and horizontal dimension), alternating every trial.

This design allowed us to answer the first question by comparing the CSE between prime→target and target→prime trials. If the CSE is larger for prime→target relative to target→prime trials, this speaks in favor of distractor-based control. However, if the CSE is of similar size in both conditions or even larger for target→prime relative to prime→target trials, this would provide evidence against distractor-based control accounts.

To answer the second question, we manipulated whether the order of prime and target repeats (e.g., previous trial: prime→target; current trial: prime→target) or switches (e.g., previous trial: prime→target; current trial: prime→target) across two trials. We assume that selection by temporal order is possible because participants use temporal order selection by default and implicitly form a task-set that includes relational information about the temporal order in which the prime and target appear. According to the literature on task switching, changing task-sets form the previous to the current trial should impair performance, while repeating the same task-set across two trials should facilitate performance. Thus, the CSE should be larger for order repetitions relative to order switches.

## Method

Raw individual data and analysis scripts can be found on the Open Science Framework *https://osf.io/c3dyf/*.

### Participants

We collected no pilot data for this task and therefore had no indication of the hypothesized effect size. For practical reasons, we assumed an effect size of *d* = .5 for the predicted modulation of the CSE. Previous research from our labs (Dignath et al., 2019; Berger, Mitschke, Dignath, Eder, & van Steenbergen, 2020; Schroeder, Dignath, & Janczyk, 2018) showed medium-to-large effect sizes for the CSE using similar tasks. According to a power analysis using G*Power (Faul, Erdfelder, Lang, & Buchner, 2007), a sample size of N = 34 was necessary to detect such an effect in a within-design (using the t-test function for the CSE difference scores, with α = .05 and 1-ββ = .8, two-sided). For counterbalancing, we planned with N = 40. In Exp. 1a, 43 volunteers (28 women, *M* = 26.09 years; range: 19–53) participated either for course credit or received 8 €, in Exp. 1b, 62 volunteers participated and were recruited via the online platform Prolific (Palan & Schitter, 2018) and received 5 £. In Exp. 1a and 1b, respectively, one participant with more than 50% errors was identified as an outlier (random responding in a two-alternative force-choice task). From the remaining sample, all participants with a mean error rate above 3SDs of all other participants were treated as outliers. In Exp. 1a and 1b, respectively, one participant (Exp. 1a: *M* = 42%, final sample *M* = 13.3%, *SD* = 6.6% errors; Exp. 1b: *M* = 45%, final sample *M* = 9.5%, *SD* = 6.7% errors) was excluded based on these criteria.

### Stimuli

In Exp. 1a, stimulus presentation and response-data collection were controlled by E-Prime (version 2.0.10.353; Schneider, Eschman, & Zuccolotto, 2002) on a 24-inch color monitor (1024 × 768 pixel (px), 144 Hz). In Exp. 1b, stimulus presentation and response data collection were controlled by the JavaScript library jsPsych (version 6.1.0.; de Leeuw, 2015). Exp. 1b was run in a browser on the private devices of the participants (the resolution of the available browser window varied between 1280 × 680 px and 2560 × 1440 px). Therefore, the visual angle of the displayed stimuli varied between the participants due to online testing and the reported visual angle refers to a situation as in Exp. 1a.

At the beginning of each block, a fixation cross (Exp. 1a: 0.48° × 0.48°) appeared in the middle of the screen (Exp. 1a: 2000 ms; Exp. 1b: 1800 ms). Each trial started with a fixation cross appearing for 200 ms. In Prime è Target trials, task-irrelevant distractor information was presented before the target, while in Target è Prime trials, the target preceded the distractor information. The program presented the prime and target (Exp. 1a: 1.15° × 1.15°) in white against a black background for 133 ms and separated by a blank screen for 33 ms. Arrows pointing to the left, right, up or down, served as primes. Target stimuli were the letters D, G, H, or S. Participants responded to the identity of the target letter by pressing the arrow keys on a QWERTZ keyboard. They were instructed to press the left and the right arrow key with the middle and index finger of the left hand and the down and up arrow key with the index and middle finger of the right hand. In case of an incorrect or missing response (within a response window of [Exp. 1a: 1500 ms; Exp. 1b: 1334 ms] after target onset), a red screen (200 ms) indicated an error. A trial ended with a blank screen (Exp. 1a: presented until the total trial duration of 2215 ms was reached; Exp. 1b: presented for 1000 ms).

### Design

RT and error rates were measured as dependent variables and we considered current congruency, previous congruency, current stimulus order, and previous stimulus order as independent variables. The present research focused on the CSE as a behavioral index of control. The CSE is modulated by several factors that are often not considered to reflect control (e.g., Davelaar & Stevens 2009; Hommel et al., 2004; Mayr et al., 2003, but see Frings et al., 2020). For instance, to avoid feature binding effects, we divided stimuli and responses into two sets of independent 2-AFC tasks that alternated every trial. Prime and target stimuli were always from the same set to avoid negative priming. To control for contingency learning, each target was preceded equally often by incongruent and congruent distractors. Within each block of trials, prime→target and target→prime trials were presented equally often with congruent and incongruent stimulus combinations in a pseudo-random order. First-order trial sequences were counterbalanced (Exp.1a: using custom MATLAB scripts (The Mathworks, Inc.); Exp. 1b: using custom JavaScript algorithms). More specifically, this counterbalancing considered all possible congruency transitions (e.g., previous trial: congruent, current trial: congruent) separately for all possible stimulus order transitions (e.g., previous trial: prime→target; current trial: target→prime) to produce an even distribution of all possible combinations across each run of 64 trials. The target stimulus-key mapping (e.g., Stimulus “S” with the left arrow key) and the assignment of stimulus-to-set (Stimulus “S” and “G” for left and right arrow keys, “D” and “G” for up and down arrow keys) were counterbalanced across participants.

### Procedure

Participants gave informed written consent before the experiment. In Exp. 1a, they were tested in individual testing rooms with a viewing distance of approximately 60 cm to the monitor. In Exp. 1b, participants were tested online, i.e., they were forwarded from the Prolific website to a website running on a server of the psychology department of University Freiburg on which the experiment files were located. All instructions were presented on the screen and both speed and accuracy were emphasized. Participants started with 8 practice trials in which only prime arrows were presented and participants responded to the prime, followed by 32 practice trials of the main task with responses to the target. The main experiment consisted of 32 blocks with 32 trials each, with self-paced breaks between every block. At the end of the experiment, participants stated whether they used the fingers of their left and right hand as instructed.

## Results

We discarded practice trials, the first trial in each block, and post-error trials (Exp. 1a: 12.8%; Exp. 1b: 9.4%) from all analyses and trials with erroneous responses (Exp. 1a: 12.6%; Exp. 1b: 9.3%) and RTs that exceeded more than 3 SDs from the cell mean for each condition (Exp. 1a: 0.9%; Exp. 1b: 0.8%) from the RT analysis. We analyzed mean RTs and error rates with a repeated-measures ANOVA with the factors current congruency [congruency, incongruent], previous congruency [congruency, incongruent], current stimulus order [target→prime, prime→target], previous stimulus order [target→prime, prime→target]. The significance criterion was set to *p* < .05 for all analyses. Standardized effect sizes (Cohen’s dz and η_p_^2^) are reported when appropriate. RT means for the reported analysis were calculated based on an average of 47 (Exp. 1a: *SD* = 7.47) or 49 (Exp. 1b: *SD* = 7.46) observations per condition.

### Experiment 1a

#### Mean RT

All main effects were significant. First, there was a main effect of current congruency, *F*(1, 40) = 78.60, *p* < .001, η_p_^2^ = .66, because RTs were longer in incongruent (*M* = 605 ms) than in congruent trials (*M* = 576 ms). Second, there was a main effect of previous congruency, *F*(1, 40) = 18.16, *p* < .001, η_p_^2^ = .31, with longer RTs following an incongruent (*M* = 595 ms) relative to an congruent (*M* = 586 ms) trial, indicating post-conflict slowing (Verguts, Notebaert, Kunde, & Wühr, 2011). Third, there was a main effect of current stimulus order, *F*(1, 40) = 411.90, *p* < .001, η_p_^2^ = .91, because RTs were longer in target→prime trials (*M* = 619 ms) relative to prime→target trials (*M* = 551 ms). And fourth, there was a main effect of previous stimulus order, *F*(1, 40) = 8.80, *p* = .005, η_p_^2^ = .18, with shorter RTs following target→prime trials (*M* = 583 ms) relative to prime→target trials (*M* = 598 ms).

There was a two-way interaction between current and previous stimulus order, *F*(1, 40) = 4.99, *p* = .031, η_p_^2^ = .11, indicating ‘switch costs’ if the order of prime and target changed from one trial to the next (Δ = 7 ms), although closer inspection of the descriptive data pattern showed that this effect was mostly limited for current target→prime trials, but not prime→target trials (see ***Table 1***).

**Table 1:** Mean RTs (in ms), error rates (in %) and CSEs for current and previous stimulus order and for current congruency and previous congruency (rows) seperated by experiment (columns).


EXPERIMENT:		1A			1B	
		
TRIAL TYPE		RT (MS)	ERR (%)		RT (MS)	ERR (%)

Target→prime following Target→prime						

Congruent following congruent		611	7.4		685	7.0

Incongruent following congruent		633	14.4		697	9.2

Congruent following incongruent		616	8.2		689	7.3

Incongruent following incongruent		640	14.2		702	8.4

CSE		–3	1.1		2	1.1

Prime→target following target→prime						

Congruent following congruent		526	10.1		730	9.5

Incongruent following congruent		566	15.5		769	12.8

Congruent following incongruent		538	11.8		739	9.3

Incongruent following incongruent		568	15.6		776	7.3

CSE		9	1.6		2	1.2

Target→prime following prime→target						

Congruent following congruent		621	9.0		695	7.0

Incongruent following congruent		639	13.0		718	9.0

Congruent following incongruent		624	8.1		704	6.1

Incongruent following incongruent		655	11.6		720	8.7

CSE		–14	0.5		6	–0.6

Prime→target following prime→target						

Congruent following congruent		529	10.9		723	9.0

Incongruent following congruent		567	15.7		768	12.6

Congruent following incongruent		545	10.4		744	8.9

Incongruent following incongruent		570	13.8		767	11.7

CSE		13	1.5		22	0.8


*Note*: The CSE was calculated for RTs and error rates as: (Previous Congruent: Current Incongruent – Current Congruent) – (Previous Incongruent: Current Incongruent – Current Congruent).

**Figure 1:**
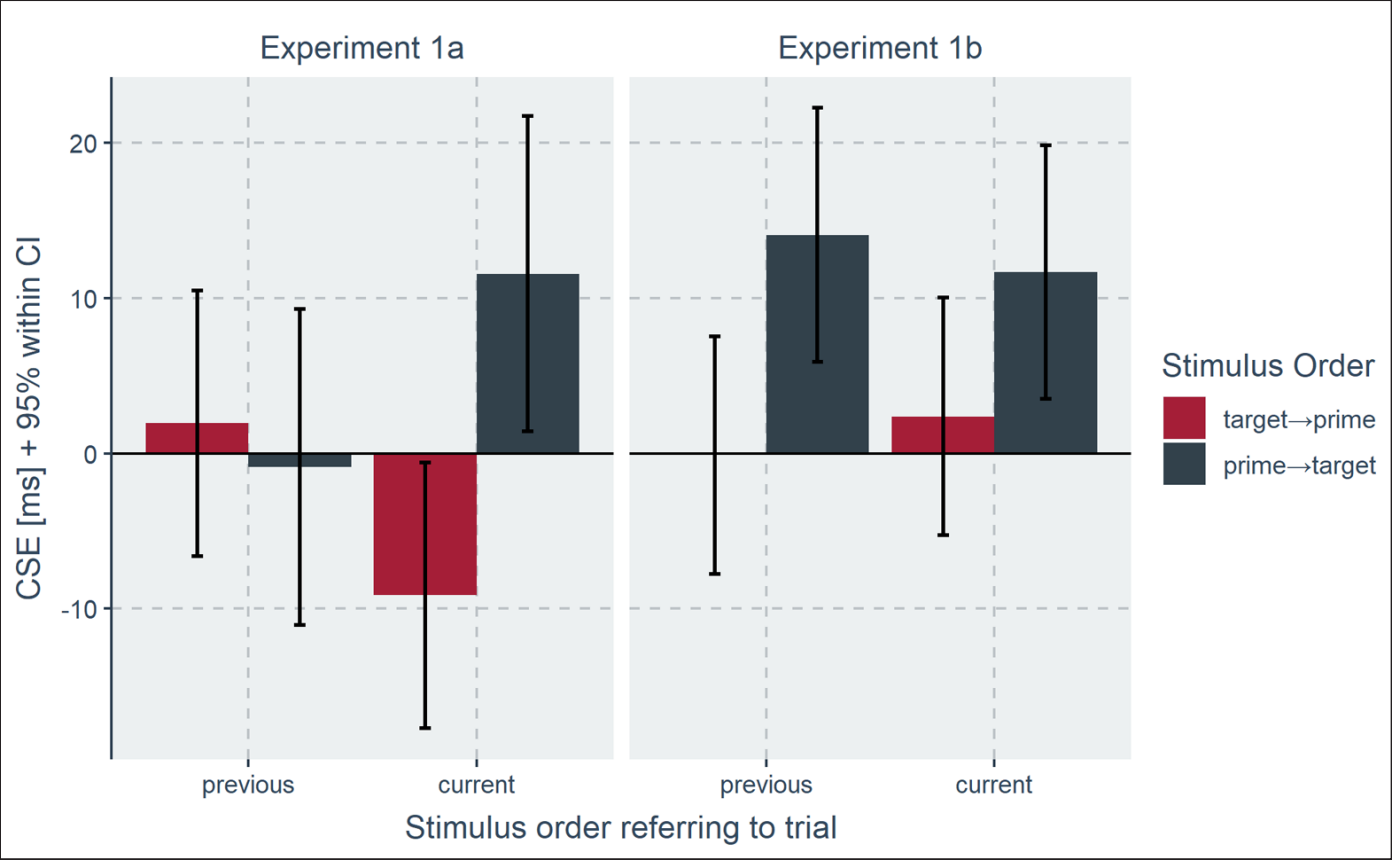
CSEs from Exp. 1a (left panel) and Exp. 1b (right panel) for target→prime (in red) and prime→target trials (in grey) separately for previous and current trials on the *x*-axis. Error bars indicate the 95% within confidence interval.

Finally, there was also a three-way interaction between the factors current congruency, previous congruency and current stimulus order, *F*(1, 40) = 7.46, *p* = .009, η_p_^2^ = .16, see ***Figure 1***, left panel. To better understand this interaction, we computed follow-up ANOVAs separately for the factor current stimulus order. For current target→prime trials, the interaction between current congruency and previous congruency was not significant, *F*(1, 40) = 2.61, *p* = .114, η_p_^2^ = .06. However, for current prime→target trials, the interaction between current congruency and previous congruency was significant, *F*(1, 40) = 6.08, *p* = .018, η_p_^2^ = .132. The CE was reduced following previous incongruent trials (Δ = 28 ms) relative to previous congruent trials (Δ = 38 ms), indicating a CSE.

#### Mean Error rates

There were three significant main effects. First, the main effect of current congruency, *F*(1, 40) = 48.64, *p* < .001, η_p_^2^ = .549, because error rates were increased in incongruent (*M* = 14.2%) than in congruent trials (*M* = 9.5%). Second, there was a main effect of current stimulus order, *F*(1, 40) = 20.93, *p* < .001, η_p_^2^ = .344, because error rates were smaller for current target→prime trials (*M* = 10.7%) relative to current prime→target (*M* = 13.0%) trials. Third, the main effect for previous stimulus order was significant, *F*(1, 40) = 5.53, *p* = .024, η_p_^2^ = .121. Trials following target→prime trials were subject to higher error rates (*M* = 12.1%) than trials following prime→target trials (*M* = 11.6%).

Further there were two significant two-way interactions. First, the interaction between current congruency and previous stimulus order was significant, *F*(1, 40) = 6.25, *p* = .017, η_p_^2^ = .17, indicating a reduced CE in trials following prime→target trials compared to target→prime trials (Δ = 1.65%). Second, the interaction between previous congruency and previous stimulus order, *F*(1, 40) = 4.80, *p* = .034, η_p_^2^ = .107, showing that the influence of previous congruency was smaller if the corresponding stimulus order was prime→target compared to target→prime (Δ = 1.71%). No other effect was significant.

### Experiment 1b

#### Mean RT

All four main effects were significant. First, the main effect for congruency was significant, *F*(1, 59) = 109.22, *p* < .001, η_p_^2^ = .649, because RTs were slower in incongruent (*M* = 740 ms) than congruent trials (*M* = 714 ms). Second, the main effect for previous congruency was significant, *F*(1, 59) = 22.68, *p* < .001, η_p_^2^ = .278, because RTs in trials following incongruent trials were slower (*M* = 730 ms) compared to trials following congruent trials (*M* = 723 ms). Third, there was a main effect for current stimulus order, *F*(1, 59) = 131.68, *p* < .001, η_p_^2^ = .691, since participants responded slower in prime→target (*M* = 752 ms) relative to target→prime trials (*M* = 701 ms). And fourth, a significant main effect for previous stimulus order was observed, *F*(1, 59) = 20.65, *p* < .001, η_p_^2^ = .259, with slower responses in trials following prime→target trials (*M* = 730 ms) compared to trials following target→prime trials (*M* = 723 ms).

Three two-way interactions were significant. First, the interaction between current congruency and previous congruency was significant, *F*(1, 59) = 7.01, *p* = .010, η_p_^2^ = .106, indicating that CEs were smaller following incongruent compared congruent trials (Δ = 7 ms). Second, there was an interaction between current congruency and current stimulus order, *F*(1, 59) = 16.49, *p* < .001, η_p_^2^ = .218, indicating that current target→prime trials showed a smaller CE than current prime→target trials (Δ = 20 ms). Third, there was an interaction between current stimulus order and previous stimulus order, *F*(1, 59) = 29.09, *p* < .001, η_p_^2^ = .330, indicating a ‘switch costs’ if the order of prime and target changed from one trial to the next (Δ = 9 ms). As in Exp. 1a, closer inspect of the data pattern showed that this effect was mostly limited for current target→prime trials (Δ = 16 ms), but not prime→target trials (Δ = –3 ms, see ***Table 1***).

Finally, there was a three-way interaction between current congruency, previous congruency and previous stimulus order, *F*(1, 59) = 6.56, *p* = .013, η_p_^2^ = .100 (see ***Figure 1***, right panel). Two follow-up ANOVAs were calculated separately for the factor previous stimulus order (target→prime; prime→target). For previous target→prime trials, no interaction emerged, *F* < 1. However, for previous prime→target trials the interaction between current congruency and previous congruency was significant, *F*(1, 59) = 11.13, *p* = .001, η_p_^2^ = .159, indicating a CSE (Δ = 14 ms).

#### Mean Error rates

There were two significant main effects. First, there was a main effect for current congruency, *F*(1, 59) = 34.37, *p* < .001, η_p_^2^ = .368, because in incongruent trials (*M* = 10.4%) the error rate was higher than in congruent trials (*M* = 8.0)%. Second, the main effect for current stimulus order was significant, *F*(1, 59) = 34.83, *p* < .001, η_p_^2^ = .371, since in prime→target trials (*M* = 10.7%) a higher error rate was observed than in target→prime trials (*M* = 7.8%). No other effect was significant.

### Combined Analysis of Experiment 1A and 1B

After analyzing both data-sets individually according to our analysis plan, we decided to perform a combined analysis of both data-sets to provide a better powered test of the hypothesis that participants utilize temporal order control. While not apriori planned, this analysis is highly constrained by closely adhering to our initial ‘temporal control as default’ hypothesis. Recall that this hypothesis predicts larger CSEs for stimulus order repetitions compared to stimulus switches. To increase the number of observations per cell, we collapsed data across the factors current stimulus order and previous stimulus order, merging data points into a single factor that coded whether stimulus order changes or repeats from previous to current trials. We analyzed mean RTs and error rates with a mixed ANOVA with the within-subject factors current congruency [congruent, incongruent], previous congruency [congruent, incongruent] and stimulus order transition [change, repeat] and the between-subjects factor Experiment [Exp.1a, Exp. 1b]. Exclusion criteria were identical to the individual analysis.

#### Mean RT

All main effects were significant. First, there was a significant main effect for current congruency *F*(1, 99) = 182.07, *p* < .001, η_p_^2^ = .648, with slower RTs in incongruent (*M* = 672 ms) relative to congruent (*M* = 645 ms) trials. Second, there was a main effect for previous congruency, *F*(1, 99) = 39.68, *p* < .001, η_p_^2^ = .286, because trials following incongruent trials were slower (*M* = 662 ms) than trials following congruent trials (*M* = 655 ms). Third, there was a main effect for stimulus order transition, *F*(1, 99) = 26.76, *p* < .001, η_p_^2^ = .213, because stimulus order switch trials were slower (*M* = 662 ms) than stimulus order repetition trials (*M* = 655 ms). Fourth, the main effect for experiment was significant, *F*(1, 99) = 58.64, *p* < .001, η_p_^2^ = .372, because RTs in Exp. 1b were slower (*M* = 726 ms) than in Exp. 1a (*M* = 591 ms).

There were 2 two-way interactions. First, the interaction between current congruency and stimulus order transition was significant, *F*(1, 99) = 4.19, *p* = .043, η_p_^2^ = .041, indicating that the CE was smaller in trials that repeated the previous stimulus order than in those where stimulus order switched (Δ = 5 ms). Second, there was an interaction between stimulus order transition and experiment, *F*(1, 99) = 9.25, *p* = .003, η_p_^2^ = .085, showing that ‘switch costs’ were smaller in Exp. 1a than in Exp. 1b.

**Figure 2:**
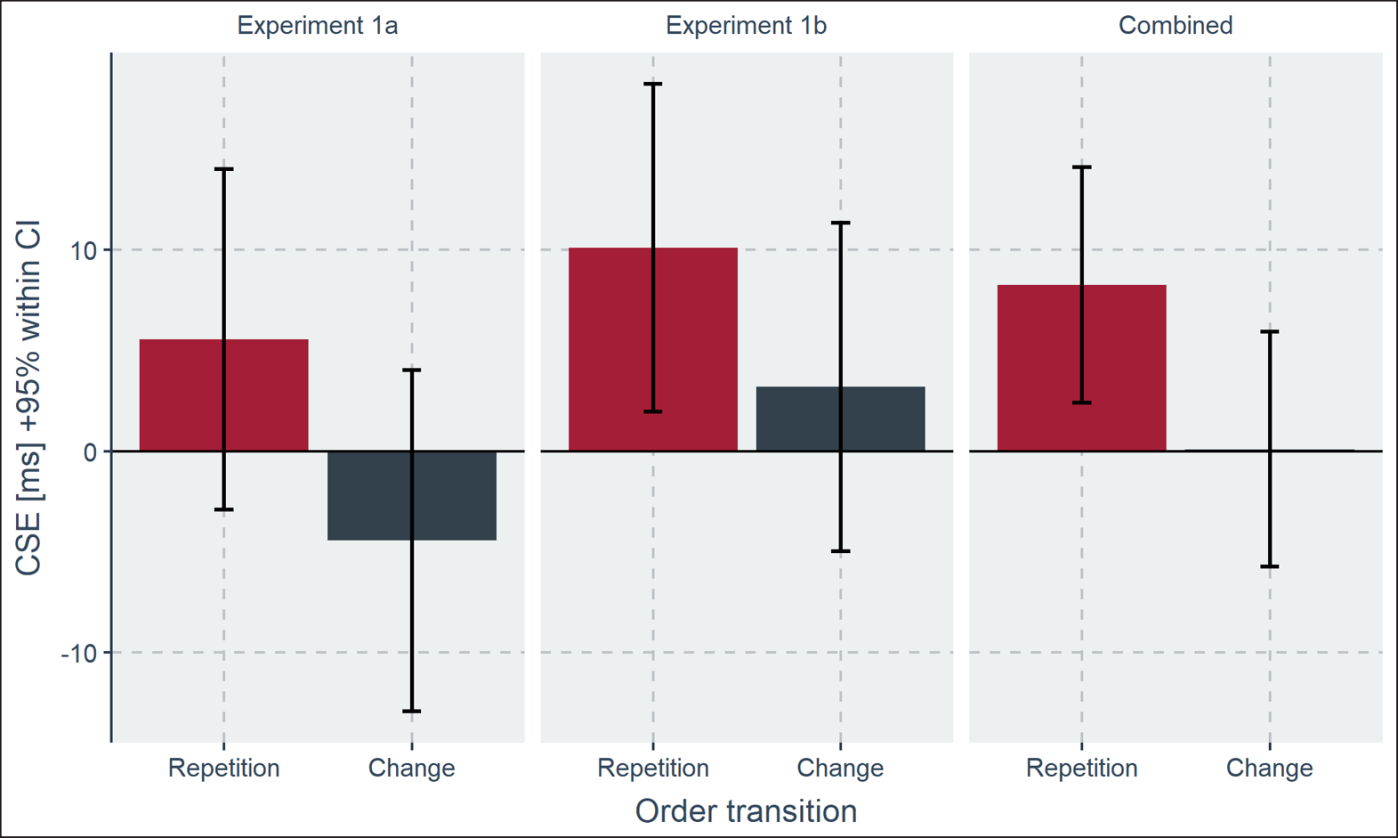
CSEs from Exp. 1a (left panel), Exp. 1b (middle panel) and combined data (right panel) as a function of stimulus order transition (repetition [in red] vs. change [in grey] of stimulus order across trials) on the *x*-axis. Error bars indicate the 95% within confidence interval.

Finally, there was a three-way interaction between current congruency, previous congruency and stimulus order transition, *F*(1, 99) = 3.94, *p* = .050, η_p_^2^ = .038 indicating larger CSEs for stimulus order repetitions compared to changes of stimulus order across trials (Δ = 8 ms; see ***Figure 2***, right panel). To investigate this interaction, we calculated two follow-up ANOVAS separately for stimulus order repetitions and changes. For stimulus order repetitions, the interaction between current and previous congruency was significant, *F*(1, 99) = 6.19, *p* = .014, η_p_^2^ = .059, indicating the expected CSE (Δ = 8 ms). For stimulus order changes, the interaction was not significant, *F* < 1. For completeness, ***Figure 2*** also presents CSEs for stimulus order repetition and changes separately for each Experiment (left and middle panel).

#### Mean Error rates

There were two significant main effects. First, the main effect for current congruency was significant, *F*(1, 99) = 89.99, *p* < .001, η_p_^2^ = .476 because participants made more errors in incongruent trials (*M* = 12.4%) than in congruent trials (*M* = 8.8%). Second, there was a main effect for experiment, *F*(1, 99) = 4.50, *p* = .036, η_p_^2^ = .043, because participants committed more errors in Exp. 1a (11.9%) than in Exp. 1b (9.3%).

Also, there was a two-way interaction between current congruency and experiment, *F*(1, 99) = 9.50, *p* = .003, η_p_^2^ = .088, indicating a smaller CE in Exp. 1b than in Exp. 1a (Δ = 2.3%). No other effect was significant.

## General Discussion

The present study tested two candidate mechanisms of control in the prime-target task. First, we asked whether control modulates irrelevant information of the prime or relevant information of the target. Second, we asked whether control operates by selection of stimulus features or by selection of temporal order of events. Regarding the first question, results provided support for accounts that explain the CSE in the prime-target task in relation to control of the prime rather than control of the target. Regarding the second question, a combined analysis of both experiments found evidence in line with the idea that participants engage control mechanisms exploiting the temporal order of events. In the following, we will discuss these findings and their implications of current theorizing on the CSE in the prime-target task and for our understanding of how control minimizes interference in general.

### CSE in the Prime-Target Task Is Due to Control of Irrelevant Prime Information

Previous studies showed that primes that precede the target increase the size of the CSE, suggesting that a central locus of control in the prime-target task is the modulation of irrelevant distractor information (Weissman et al., 2015). However, it remained unclear whether this effect was due to prime-based control, or whether it reflects the contribution of an additional control mechanism that modulates prime and target information according to their temporal order (Hazeltine et al., 2011). Critically, in the comparison condition in Weissman et al. (2015), primes and targets were presented simultaneously, a situation in which this temporal control mechanism is not possible. The present study controlled for this possible confound by consistently presenting the two stimuli sequentially and varying the order of prime and target. In both experiments, the CSE was larger for prime→target trials relative to the target→prime trials. This finding provides further evidence for the view that, in the prime-target task, control modulates distracting information of the prime and not the target (Ridderinkhof, 2002; Weissman et al., 2015). In addition, Weissman et al. (2015) reported larger CSEs both for current and previous prime→target trials. The present results are largely compatible with this finding. While Exp. 1a showed significantly larger CSEs for current prime→target trials, Exp. 1b showed significantly larger CSEs for previous prime→target trials. Together, results from Weissman et al. (2015) and the current study suggest that the presentation of the prime before the target is important both for applying control (e.g., an effect in the current trial) and for triggering control (e.g., an effect in the previous trial).

It remains unclear why Exp. 1a found larger CSEs selectively for current prime→target trials (see ***Figure 1***, left panel). One possible explanation could be due to changes in the decision criterion. As indicated by significant main effects of current order pointing in opposite directions for RT and error rates (see Bombeke, Langford, Notebaert, & Boehler, 2017, for similar findings), participants might have favored faster responses for prime→target trials at the cost of increased error rates. Previous research has shown that speed instructions boost the CSE (van Veen, 2006), suggesting that distractor-based control in the current study could be achieved by dynamic changes in the speed-accuracy trade-off. Interestingly, there was no indication of such a speed-accuarcy trade-off in Exp. 1b, which showed numerically larger CSEs for both current and previous prime→target trials (see ***Figure 1***, right panel). Although such a speed-accuracy explanation is not incompatible with a view of the CSE as a marker of control, future research could use computational modeling like the drift-diffusion model to get a more complete account how control modulates responding in prime→target trials (e.g., via changes in the speed of evidence accumulation or changes in the response criterion).

Although the present results support response-related accounts which ascribe the prime a critical role for control (Ridderinkhof, 2002; Weissman et al., 2015), they cannot differentiate whether control modulates motor activation of the response instigated by the prime, S-R translation triggered by the prime or perceptual activation of prime encoding. For instance, Wendt and colleagues (2014) have shown that different proportions of congruent to incongruent trials modulate early visual activity related to the prime, as indicated by EEG. Further research could use different EEG components to arbitrate how different stages of prime processing contribute to the CSE in the prime-target task. Finally, although the CSE in the present study could be attributed to prime-based control, we do not deny that target-based control processes can also contribute to adaptative performance. Indeed, a series of studies found evidence for fast control processes that modulate target activity within a trial (Scherbaum, Fischer, Dshemuchadse, & Goschke, 2011; Pastötter, Dreisbach, & Bäuml, 2013; Nigbur Schneider, Sommer, Dimigen, & Stürmer, 2015). Certainly, it would be interesting to get a better understanding of how control operates on different timescales and how different control mechanisms are geared to each other.

### CSE in the Prime-Target Task Is Due to Temporal Order Selection

Regarding the second question, whether control in the current task utilizes only information within the stimulus (perceptual features) or considers also relational information between stimuli (temporal order), a combined analysis of both Exp. 1a and 1b showed a significant larger CSE for stimulus order repetitions (e.g., previous trial: prime→target; current trial: prime→target) compared to changes (e.g., previous trial: prime→target; current trial: target→prime). This suggests that participants formed a task-set that specified temporal information about the order of prime and target stimuli in combination with a relative weighting of prime and target information according to the congruency level. Repeating the same stimulus order on the next trial allows the application of this task-set and thus increases control, as indicated by larger CSEs, compared to changing of stimulus order. This finding extends previous research showing temporal order selection for instructed (explicit) task-sets (Hazeltine et al., 2011) by demonstrating that participants form and use implicit task-sets (see Dreisbach et al., 2006). Interestingly, participants harnessed temporal order selection despite instructions and stimuli that afforded a selection based on perceptual features. We interpret this as evidence in favor for a ‘temporal control as default’ hypothesis, suggesting that in the prime-target task, temporal order information has a privileged status.

A next step requires a better characterization of possible boundary conditions to understand in more detail when and how temporal information guides control. For instance, one limitation could be due to the stimulus material used, with prime stimuli (e.g., arrows) that were composed of a different stimulus category than targets (e.g., letters), and which were thus clearly distinguishable from target stimuli based on their perceptual features (e.g., arrows vs. letters) and also differed in their S-R translation. Arguably, it might have been easier for participants to engage in feature-based selection with primes that are easy discriminable from targets and which consequently rendered temporal selection less likely. Another reason could be that frequent and unpredictable order switches of prime and target hampered the utility of temporal order selection. Consider a trial sequence in which prime-target order switched (e.g., previous trial: prime→target, current trial: target→prime). Here, temporal order selection would actually be disadvantageous, since it would reduce attention to the target, but increase attention to the prime in the current trial. And finally, instructions in the present study asked participants to select targets according to perceptual features which rendered temporal selection nominally irrelevant. Together, these factors might have limited temporal order selection relative to feature-based selection. Future research could test this conjecture more directly i.) by manipulating similarity between prime and target stimuli, by ii.) changing utility of temporal selection (e.g., by comparing blocks of trials with fixed against random order) and iii.) by testing different instructions that emphasize temporal over feature-based selection.

The present modulation of the CSE by stimulus order transition reflects a selection of prime and target information based their relative order. Such a post-perceptual selection mechanism is incompatible with the conflict monitoring account of the CSE, which describes control as a change of pre-attentive perceptual features (e.g., color or location; see Botvinick et al., 2001). However, it is compatible with an emerging view that describes the CSE in terms of binding and retrieval of episodic memory (Dignath, Johannsen, Hommel, & Kiesel, 2019; Frings et al., 2020; see also Schumacher & Hazeltine, 2016). According to this perspective, in each trial, participants store various events in memory. This memory includes information about concrete, observable events like stimuli, responses and context features, but also more abstract events like the task-set employed (see Egner, 2014). Repetition of any of these elements (e.g., a context feature) in the next trial retrieves associated information from memory and thus reinstates the previous task-set. Mounting evidence supports this notion, showing that abstract mental-states like a task-set can be retrieved from one trial to the next (e.g., Dignath et al., 2019; Grant, Cookson, & Weissman, 2020; Jiang, Brashier, & Egner, 2015; Giesen & Rothermund, 2014; Singh, Frings, & Moeller, 2019; Nett, Bröder, & Frings, 2016). The present results extend this view by suggesting that task-sets which control prime and target activity according to their relative temporal order can come under mnemonic control, so that reencountering the same stimulus triggers an automatic retrieval of the previous task-set.

Interestingly, it has been debated whether event-files can include temporal information (see Hommel, 2009). Empirical studies reported mixed results, with some studies showing no effect of temporal information on bindings (Moeller & Frings, 2019), while others found supportive evidence showing that temporal information is bound and retrieved in event-files (Bogon, Thomaschke, Dreisbach, 2017). The present results weigh in favor for the latter view, suggesting that under some circumstances (e.g., when the structure of the task renders temporal information salient), temporal information can be bound and retrieved in event-files. This is in line with a theoretical account that highlights a critical role of temporal information for the CSE in general (Schmidt, 2013; Schmidt & Weissman, 2016). Indeed, timing research suggests that participants readily learn temporal information reflecting the point in time when to respond which biases response timing in subsequent trials (e.g., Dignath, Pfister, Eder, Kiesel, & Kunde, 2014; Grosjean, Rosenbaum, & Elsinger, 2001). According to this perspective, the CSE indicates rather a rhythmic bias due to temporal learning than a change in control (Schmidt & Weissman, 2016). It seems reasonable to assume that temporal learning of response times depends on the order of distractor and target which could provide critical context information for timing. Although this rhythmic bias account is not incompatible with the binding and retrieval account (e.g., Schmidt, De Houwer, & Rothermund, 2016), the present procedure of changing temporal order of distractor and target across trials might be an interesting tool to test specific predictions of the temporal learning account.

## Summary

This research addressed the structure of control in the prime-target task. By controlling for differences in attentional selection (e.g., selection by feature vs. selection by temporal order), the study provided more direct evidence for the claim that control is exerted over irrelevant prime information rather than relevant target information. This observation supports theoretical accounts suggesting that control over distractor information is a critical determinant of the CSE in the prime-target task. In addition, the study indicates that in sequential tasks, like the prime-target task, participants may use temporal order information by default to guide attentional selection. This observation supports binding and retrieval accounts that assume that task-sets come under mnemonic control. In sum, this study shows that CSEs in the prime-target task are the result of multiple control mechanisms.

## Data Accessibility Statement

Raw individual data and commented analysis scripts can be found on the Open Science Framework, *https://osf.io/c3dyf*. DOI *10.17605/OSF.IO/C3DYF*.
